# Nanoporous CREG-Eluting Stent Attenuates In-Stent Neointimal Formation in Porcine Coronary Arteries

**DOI:** 10.1371/journal.pone.0060735

**Published:** 2013-04-03

**Authors:** Jie Deng, Yaling Han, Mingyu Sun, Jie Tao, Chenghui Yan, Jian Kang, Shaohua Li

**Affiliations:** 1 Department of Cardiology, Institute of Cardiovascular Research of People’s Liberation Army, Shenyang Northern Hospital, Shenyang, Liaoning, China; 2 Division of Vascular Surgery, Department of Surgery, Robert Wood Johnson Medical School-UMDNJ, New Jersey, United States of America; Cliniche Humanitas Gavazzeni, Italy

## Abstract

**Background:**

The goal of this study was to evaluate the efficacy of a nanoporous CREG-eluting stent (CREGES) in inhibiting neointimal formation in a porcine coronary model.

**Methods:**

In vitro proliferation assays were performed using isolated human endothelial and smooth muscle cells to investigate the cell-specific pharmacokinetic effects of CREG and sirolimus. We implanted CREGES, control sirolimus-eluting stents (SES) or bare metal stents (BMS) into pig coronary arteries. Histology and immunohistochemistry were performed to assess the efficacy of CREGES in inhibiting neointimal formation.

**Results:**

CREG and sirolimus inhibited in vitro vascular smooth muscle cell proliferation to a similar degree. Interestingly, human endothelial cell proliferation was only significantly inhibited by sirolimus and was increased by CREG. CREGES attenuated neointimal formation after 4 weeks in porcine coronary model compared with BMS. No differences were found in the injury and inflammation scores among the groups. Scanning electron microscopy and CD31 staining by immunohistochemistry demonstrated an accelerated reendothelialization in the CREGES group compared with the SES or BMS control groups.

**Conclusions:**

The current study suggests that CREGES reduces neointimal formation, promotes reendothelialization in porcine coronary stent model.

## Introduction

Multiple studies have shown a remarkable reduction in the rate of restenosis and the need for new revascularization procedures associated with drug-eluting stents (DES) compared with conventional bare metal stents[1], [2]. However, the long-term safety of DES is far less certain [3], [4]. Currently, the approved drug-eluting stent platforms use a polymer-based coating for the retardation of drug release. There is evidence that the application of polymers may lead to hypersensitivity reactions and, in a few cases, late cardiac death [5], [6]. Furthermore, the issue of late-stent thrombosis associated with DES, particularly after discontinuation of antiplatelet therapy, is currently the subject of ongoing discussion. By preventing the polymer-induced stimulation of the arterial wall, polymer-free DES may overcome the complications associated with DES.

Sirolimus and various sirolimus analogues have been shown to be effective against restenosis in DES formats. Sirolimus-eluting stents may also elicit adverse effects, as sirolimus pharmacology is not cell type-specific but is more generally targeted toward proliferating cells[7]. In particular, recent studies suggested that DES implantation adversely affects local endothelium regeneration [8]. While the coronary arterial stent has had a substantial clinical impact, other more difficult stent implantation sites are the current targets for new DES techniques seeking to demonstrate efficacy in challenging diabetic patients and more complex disease sites, such as small vessel and bifurcated lesions, restenosis lesions and peripheral vasculature (e.g., venous sites). Thus, new stent designs will likely be combined with new therapeutic drug mixtures.

Human CREG is a 32-kD protein that was initially identified as a transcription repressor that antagonized transcription activation and cellular transformation by the adenovirus E1A protein [9], [10]. We previously identified CREG as a markedly upregulated gene during human vascular smooth muscle cell (VSMC) differentiation upon serum withdrawal[11]. Our subsequent studies demonstrate that the overexpression of CREG in vascular SMCs induces a differentiated phenotype by enhancing the synthesis of SMC-specific marker proteins and inhibiting proliferation, migration and apoptosis [11], [12], [13], [14], [15], [16]. Furthermore, we have shown that CREG is downregulated in the balloon-injured carotid artery and that retrovirus/adenovirus-mediated CREG transfer inhibits neointimal hyperplasia after vascular injury [17], [18]. In this study, we evaluated the adsorption and elution characteristics of CREG protein using nanoporous stents and the effect of such loaded stents on endothelial cell and VSMC proliferation in a pig coronary model. We suggest that a sustained delivery of CREG protein to the vessel wall can be achieved with loaded stents, which could limit neointimal formation in vivo.

## Materials and Methods

### Ethics Statement

Ethics approval was given by the Ethics Committee of the Shenyang Northern Hospital. The experimental and animal care procedures were approved by the Institutional Animal Care and Use Committee of Shenyang Northern Hospital. The investigation conformed to the Guide for the Care and Use of Laboratory Animals published by the US National Institutes of Health (NIH Publication No. 85–23, revised 1996). All participants provide their written informed consent to participate in this study.

### Stent coating

This study utilized 15-mm nanoporous stents (Lepu Medical, China). The porosity (roughened surface) of the 316L stainless steel stent is produced by mechanical treatment/modification. The roughness of the stent surface, determined by a perthometer, ranges from 100 nm-1 µm, allowing drug deposition and prolonged drug release without the application of polymers. CREG protein was created as previously described[16] and was labeled with ^125^I by the Iodo-gen method[19]. The nanoporous stent wire segments were immersed in a solution of CREG protein (specific activity, 30 µCi/mg) diluted to 0.1, 0.5, or 1.0 mg/mL in a coating buffer (0.01 mol/L sodium phosphate/0.145 mol/L sodium chloride, pH 7.2) at 37°C. The CREG protein solutions were contained in 1.5-mL polypropylene (Eppendorf) tubes, and the wire segments were placed vertically and completely immersed in each solution. At 1, 4, 12, 24, and 48 hours after immersion, the wires were removed from each solution and rinsed 3 times with 5 mL of PBS, and the protein binding was quantified by counting the radioactivity associated with each wire. Ten wire segments were assessed at each time point for each concentration.

### CREG protein Elution from Stent Wire in Vitro

The stents were immersed in 1-mg/mL solutions of CREG protein, diluted in coating buffer, at 37°C for 24 hours as described above. The baseline CREG protein binding to the wires was ascertained by measuring the radioactivity of the wires. To assess the pharmacological release kinetics, the coated stents (n  =  3 each) were deployed ex vivo and inserted into an Eppendorf tube filled with 1 ml of PBS containing 1% BSA. The tube was maintained in constant rotation at 37°C to simulate blood flow. PBS was removed at multiple time points to quantify CREG protein levels by measuring the radioactivity of the wires. Each time PBS was removed, the stent was placed in fresh PBS. The cumulative CREG protein release was calculated.

### In vitro smooth muscle cell and Endothelial cell proliferation assays

Primary cultures of human vascular smooth muscle cells (VSMCs) were established by explant outgrowth from a segment of internal thoracic artery retrieved during coronary artery bypass surgery. Primary human umbilical vein endothelial cells (HUVECs) were isolated and purified from umbilical cords. Collections were approved by the Ethics Committee of the Shenyang Northern hospital, and informed consent was obtained from each patient. VSMCs and HUVECs were plated into sterile 48-well flat-bottom tissue culture plates at a final concentration of 1×10^5^ cells/ml and incubated at 37°C in a 5% humidified CO_2_ atmosphere. Cell proliferation was induced by mitogenic stimulation with platelet-derived growth factor (PDGF, 50 ng/ml) or endothelial cell growth factor (ECGF, 50 ng/ml) for VSMCs and HUVECs, respectively. The CREG protein-eluting stents (stents were immersed in 1-mg/mL solutions of CREG protein), BMS (Lepu Medical, China) or the polymer-coated sirolimus-eluting stents (Lepu Medical, China) were added, and cell counts were performed every 24 h for 10 days (VSMCs) or 4 days (HUVECs).

### Porcine coronary stent model

The study animals were female domestic pigs weighing 22–35 kg. All selected animals were visited three days before implants, to verify the clinical conditions. The animals were given 300 mg of acetyl salicylic acid (ASA) and a 300-mg clopidogrel loading dose (Plavix, Sanofi Aventis, Gouda, The Netherlands) 1 day prior to stenting, and they were given 100 mg of ASA and 75 mg of clopidogrel daily during the follow-up period. A 24-hour food and liquids fasting before intervention was adopted. On the day of the stent implantation, the pigs were anesthetized with ketamine (20 mg/kg intramuscularly) and xylazine (2 mg/kg intramuscularly). They received 3 L/min of supplemental oxygen continuously through an oxygen mask. After 2% subcutaneous lidocaine was administered at the cut-down site, the right femoral artery was surgically exposed and a 6F sheath was inserted. Continuous hemodynamic and surface electrocardiographic monitoring was performed throughout the procedure. After Heparin (10,000 units) was administered intravenously as a bolus, prior to the procedure, the target coronary artery was engaged using standard 6F JR guide catheters, and control angiograms of both coronary arteries were performed using a nonionic contrast agent in two orthogonal views. All the animals were fed with a standard laboratory chow diet without lipid or cholesterol supplementation in the animal laboratory center. Signs of infection, body weight, body temperature, diet, action, and the condition of incision healing were monitored throughout the study.

18 CREGES, 18 SES, and 18 BMS were deployed into the right and left coronary arteries of 18 domestic pigs (3 stents per animal), the size and length of the stents used were 2.75–3.0 and 15 mm, respectively. The stent-to-artery ratio ranged from 1.1 to 1.2, with similar ratios in all groups. Repeated angiograms were obtained immediately after the stent implantation. All of the equipment was then removed, and the femoral artery was ligated. Two weeks after stenting, 6 of the animals were euthanized by an intracoronary injection of potassium chloride, and the coronary arteries were collected for endothelialization analysis. Four weeks after stenting, the other 12 animals underwent repeated angiography in the same orthogonal views as those used before death, and an intracoronary injection of potassium chloride (20 mL) was performed. The stented artery segments were excised for biochemical, immunohistochemical, and morphometric analyses. In order to evaluate the effects of nanoporous stents (NS) without CREG on neointimal formation after stent implantation. 9 NS were implanted into the right and left coronary arteries of 3 domestic pigs (3 stents per animal). The arteries were harvested at 4 weeks and stained with hematoxylin and eosin.

### Histology and Immunohistochemistry

The stented artery segments were processed as described previously. The segments were cut into 5 pieces, each approximately 3 mm long. The sections from the proximal, distal and medial pieces were embedded in methyl methacrylate mixed with n-butyl methacrylate to allow for sectioning through the metal stent struts. The serial sections were stained with hematoxylin-eosin(HE). The neointimal area, the area within the internal elastic lamina and external elastic lamina, and the lumen area were measured by computerized morphometry. An experienced cardiovascular pathologist performed the histopathological evaluation of each artery. All images were captured using an Olympus microscope equipped with a digital camera (HC-2500) and were analyzed using image analysis software (Image-proplus 5.1, Olympus). Histopathological injury and inflammation scores of the stented aortas were evaluated according to the recommended guidelines[20].

For immunohistochemical analysis, after the stent struts were gently removed with microforceps, the tissue was dehydrated, embedded in paraffin, and cut into 5-µm-thick sections. The sections were subjected to immunostaining with antibodies against α-SMC-actin (1/2700, Sigma), macrophages (CD68, 1/300, Serotec) or Ki67 (1/100, Serotec), a nuclear antigen expressed during the late G1, S, G2 and M phases. The α-SMC-actin content of the intima and media was determined using stereological point counting and expressed as a percentage. Ki67- and CD68-positive cells were counted at four stent struts (3, 6, 9 and 12 o’clock), as recommended, and the average density (positive cells/mm^2^) was calculated. The coronary artery lumen circumference (T) and the length of the luminal border stained positive for CD31 (1/200, Sigma) (E) were measured and the endothelialization rate was calculated as ∑E/∑T×100%. For quantification of the immunohistochemical images, care was taken to select stented sites with minor injury in the neointima induced by detachment of the stent strut. Because this process of selecting sections with the least injury may introduce bias, at least 5 representative images were selected, and the percentage of immunopositive cells with respect to the total number of cells in each image was calculated. The average of the 5 images was reported for each animal.

### Scanning electron microscopy

To evaluate early endothelialization, scanning electron microscopy (SEM) was performed in other 3 pigs at 5 days. After fixation with 2.5% glutaraldehyde in 0.15 mol/l cacodylate buffer, were dehydrated, longitudinally cut, critical point dried, and finally gold coated. SEM images were acquired using a Cambridge S200 (Leo, Carl Zeiss, Oberkochen, Germany) via Pixie-3000 real time active SEM framestore (Deben Software, Suffolk, UK). Percentage of Endothelial coverage was quantified with Image Pro Plus software on five randomly chosen low magnification SEM fields per stent as previously described [21].

### Statistical analysis

Data are presented as the mean±standard deviation. Comparisons between the groups were performed by analysis of variance (ANOVA) between groups with LSD post-hoc tests, where appropriate. Probability values (p) of less than 0.05 were considered significant. Statistical analysis was performed using the SPSS statistical software package 15.0 for Windows.

## Results

### CREG protein binding to the Stent Wire

**Figure 1:**
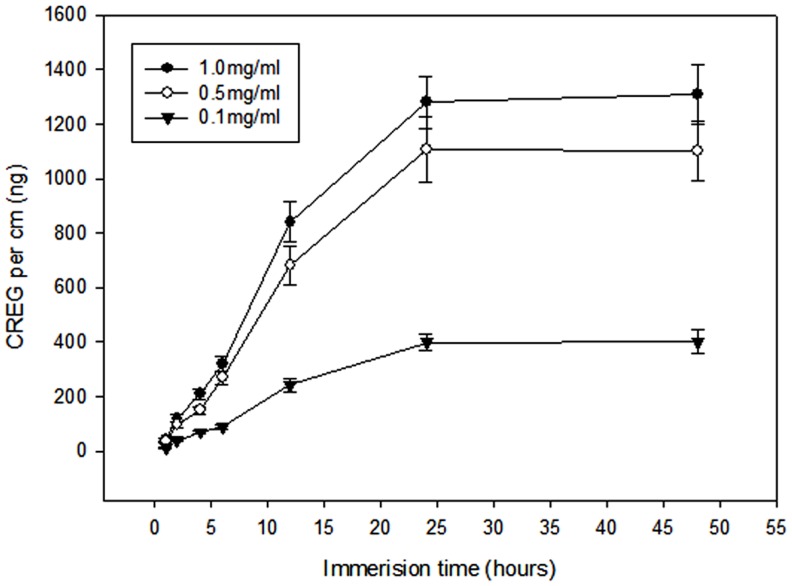
The adsorption of CREG protein on the stent wires at three different concentrations over 48 hours.

CREG protein binding to the stent wires was evaluated at three different concentrations (0.1, 0.5, or 1.0 mg/mL). There was an increase in the amount of CREG protein bound in relation to both the stent wire immersion time and the CREG protein concentration (Figure 1). More than 96% of the final amount bound at each concentration was adsorbed within 24 hours; the maximal CREG protein binding was therefore defined as the amount of agent bound to the stent wires after 24 hours of immersion in a 1.0 mg/mL solution. Using this method, the maximum CREG protein binding (per 10-mm wire) was 1280±96 ng for the 1.0 mg/mL solution, 1108±120 ng for the 0.5-mg/mL solution, and 398±30 ng for the 0.1-mg/mL solution.

### Elution of the CREG protein from the Stent Wire in Vitro

**Figure 2:**
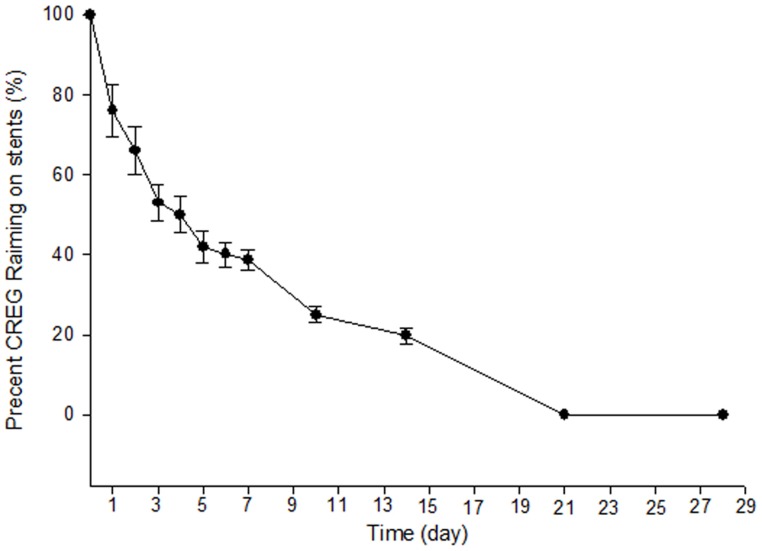
The proportion of CREG protein remaining bound to the stent. Each data point represents the mean value obtained from 12 wire segments (error bars denote ±1 SD).

The time-dependent, radioactivity-based measurement demonstrated a biexponential elution of CREG protein from the stent, with an initial rapid washoff followed by a slower exponential reduction in the amount of protein persisting on the stent wires (Figure 2). After 7 days of continuous perfusion with PBS+1% BSA, almost 35% of the amount of CREG protein originally adsorbed remained bound to the wires.

### In vitro VSMC and HUVEC proliferation assay

**Figure 3:**
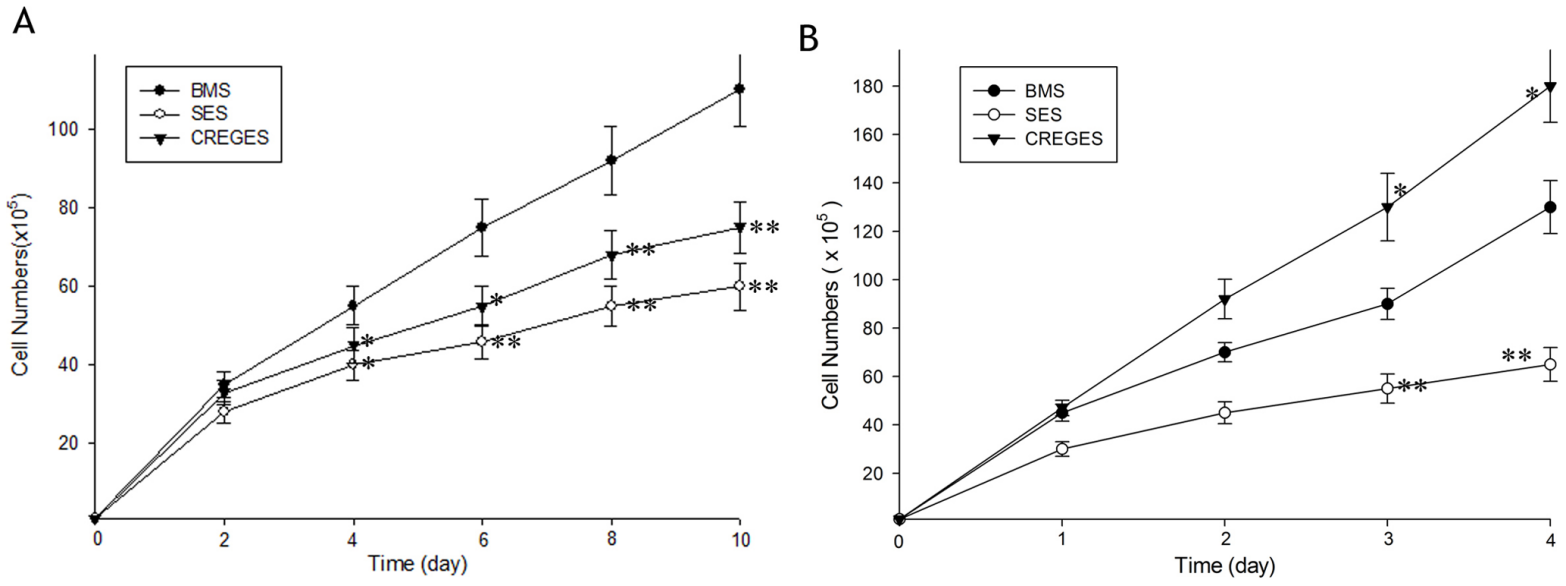
In vitro proliferation assays using human VSMCs(A) and ECs(B), stimulated with PDGF at 50 ng/ml and ECGF at 50 ng/ml, respectively. **P<0.01 and *P<0.05, the cell counts of the BMS group vs. those of the CREGES and SES groups.

Compared with the BMS, the CREGES and SES markedly inhibited VSMC growth from day 4 to day 10, as assessed by cell counts (*p*<0.05), and no significant differences between the CREGES and the SES were observed throughout the 10-day observation period (p  =  NS; Figure 3 A). Due to the rapid growth of HUVECs stimulated with ECGF in vitro and the resulting contact inhibition, observation periods beyond 4 days were not possible. On day 3 and 4, the SES significantly inhibited HUVEC growth (p < 0.01) and the CREGES increased HUVEC growth compared with the BMS (p<0.05; Figure 3 B).

### CREG-Eluting Stents Inhibit Neointimal Formation

We performed angiography immediately after stent implantation and observed no dissection, perforation, laceration of the aortic wall, intravascular thrombosis or obstruction. Angiographic follow-up after stent implantation did not show aneurysm, thrombosis, fiber deposition, or dislocation of the implanted stents. Animals in 3 groups gained an average of 4.7–5.0 kg in body weight during the course of the study, and clinically tolerated well the surgical procedures and treatments.

**Figure 4:**
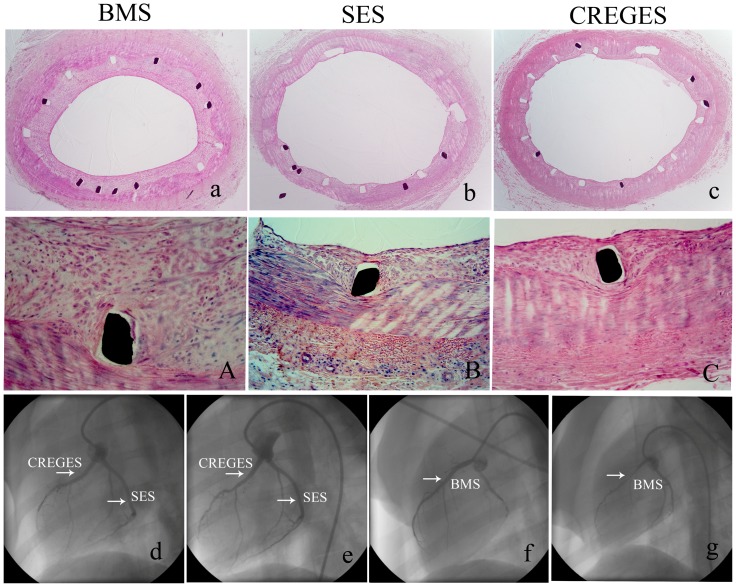
The coronary arteries of pigs implanted with BMS, SES, or CREGES. The coronary arteries were harvested 4 weeks after stent implantation, paraformaldehyde-fixed, paraffin-embedded, and stained with HE. The upper panel shows representative arteries from the BMS (a) and SES (b), and CREGES(c) groups. The middle panel shows high-magnification images of the arteries in the corresponding groups. The lower panel shows representative coronary angiographic images immediately after (d, f), and 4 weeks after stent implantation (e, g) in the BMS, SES, and CREGES groups. The white arrow indicates the site implanted. The implantation of BMS induced significant neointima formation. In contrast, the CREGES and SES significantly reduced neointima formation.

After 4 weeks, coronary diameter stenosis was significantly decreased in the CREGES group compared with the BMS group, but no difference was observed between the SES and CREGES groups. The mean neointimal area was significantly decreased in the presence of CREGES compared with BMS and nanoporous stents (NS), but no difference was observed between SES and CREGES (Figure S1). After 4 weeks, there were no significant differences in the injury or inflammatory scores between the SES, CREGES, and BMS groups (Figure 4, table 1).

**Table 1:** Morphometric analysis in 4 weeks.

Parameter	SES(n = 12)	CREGES(n = 12)	BMS(n = 12)
Lumen area(mm^2^)	2.32±0.77	2.61±0.47	1.71±0.45
Neontimal area(mm^2^)	1.61±0.60*	1.70±0.39*	3.23±1.56
Media area(mm^2^)	1.26±0.11	1.11±0.17	1.17±0.15
Percent stenosis(%)	15%±4%*	20%±3%*	48%±4%
Injury score	1.14±0.35	1.26±0.61	1.26±0.63
Inflammatory score	1.35±0.48	1.32±0.47	1.34±0.48

* P <0.05 vs. BMS.

**Figure 5:**
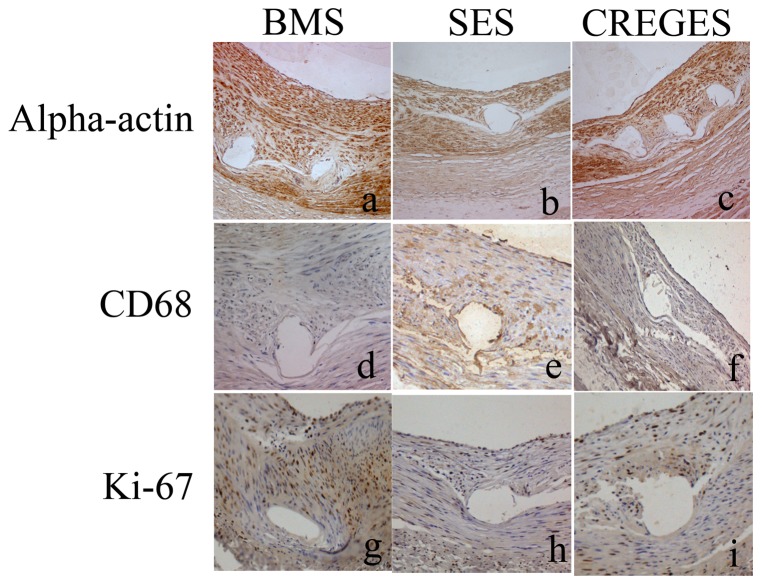
Representative photomicrographs of a-SMC actin (a, b, c), CD68(d, e, f) and Ki67 (g, h, i) in the BMS, SES, and CREGES groups. Cell replication (Ki67) occurred less frequently around the SES and CREGES struts compared with the BMS struts.

To investigate the effect of CREG on neointimal formation after vascular injury, we performed immunostaining for α-smooth muscle actin to evaluate smooth muscle cell proliferation and neointimal growth, and we stained for macrophages to evaluate the infiltration of inflammatory cells post-stent implantation. To analyze the effects of CREG on cellular proliferation, we also performed immunostaining with an antibody against Ki-67, which is a specific marker for cells in the S-phase. Immunohistochemistry showed no differences in the intimal or medial α-SMC-actin content or the CD68-stained macrophages between the stents. However, the proliferation marker Ki-67 (13±2 vs. 21±3 cells/mm^2^, P = 0.03) was reduced in the CREGES group compared with the BMS group, while no difference was observed between the SES and CREGES groups (9±2 vs. 13±3 cells/mm^2^, P = 0.62) (Figure 5).

### CREG-Eluting Stents Promote Early Vascular Endothelialization

**Figure 6:**
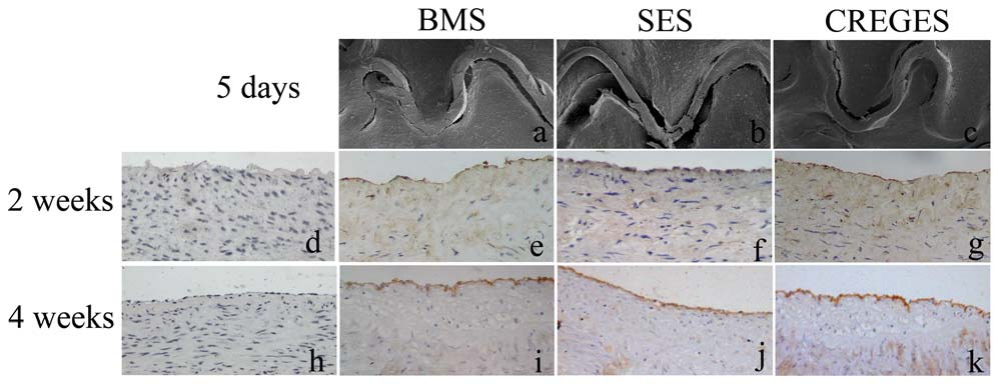
CREGES accelerate reendothelialization. SEM images (a:BMS, b:DES, c:CREGES,40X) from the porcine coronary arteries 5 day after the stents implanted. The coronary arteries were collected 2 and 4 weeks after the stent implantation. Paraffin-embedded sections were immunostained with an anti-CD31 antibody to reveal the endothelium (the brown layer covering the neointima). CREGES implantation promoted reendothelialization of the injured coronary artery to a greater extent than BMS or SES implantation at 2 weeks. Representative images of stained sections in the (e) BMS, (f) SES, and (g) CREGES groups at 2 weeks and in the (i) BMS, (j) SES, and (k) CREGES groups at 4 weeks. Negative control (d,h).

The early endothelialization of stented arteries (n  =  3 for each group) was examined using SEM at 5 day after stent implantation. CREGES stents were always covered by a higher percentage endothelium than BMS (48.3 ± 3.9% vs. 27.1 ± 3.1%, P<0.05) and SES (48.3 ± 3.9% vs. 21.2 ± 2.1%, P<0.05) (Table 2, Figure 6). We harvested coronary tissues 2 and 4 weeks after the stent implantation and immunostained the tissues for CD31, a marker of endothelial cells. We observed a nonconsecutive linear staining on the surface of the neointima in the BMS, CREGES and SES groups 2 weeks after stenting. In contrast, endothelial coverage was more continuous in the CREGES group (Figure 6). The endothelialization rates in the CREGES, BMS, and SES groups were (80.1± 5.2%), (56.2 ± 4.1%), and (54.4 ± 4.3%), respectively (P < 0.01, CREGES group vs. the other two groups). However, 4 weeks after the stent implantation, CD31 staining exhibited a consecutively linear pattern in all three of the groups, suggesting that CREGES promote accelerated endothelialization.

**Table 2:** Endothelialization analysis at 5days, 2 weeks and 4 weeks.

Parameter	SES	CREGES	BMS
5 days			
Above struts	21.2% ± 2.1%*	48.3%± 3.9%	27.1%± 3.1%*
Between	32.1% ± 2.8%*	65.4% ± 7.2%	35.7% ± 2.9%*
2 weeks	54.4% ± 4.3%*	80.1%± 5.2%	56.2%± 4.1%*
4 weeks	100%±0%	100±0%	100%±0%

* P <0.05 vs.CREGES.

## Discussion

In this study, we implanted CREGES, BMS, or SES in the coronary arteries of pigs. We found that CREGES implantation reduced neointima proliferation and accelerated vascular reendothelialization.

The effect of CREG on neointima formation has been evaluated in rat and rabbit models following vascular injury [17], [18]. We previously collected and cultured the remnants of lower limb arteries from patients undergoing amputation. Compared with the untreated control arteries, the human arterial organ cultures treated with CREG protein had reduced intimal thickness. Therefore, these data suggest that the forced expression of CREG in the artery wall after acute vascular injury can attenuate neointimal hyperplasia[15]. CREG delivery may have therapeutic potential for the prevention of restenosis. In this study, we observed a 33% reduction of neointima formation in CREGES-implanted coronary arteries compared with BMS groups. These results suggest that CREGES is a promising therapy for restenosis after percutaneous coronary angioplasty. Our Previous studies have shown potential mechanism of CREG attenuates neointimal hyperplasia. CREG promotes a mature smooth muscle cell phenotype and interacts with M6P/IGF2R and activates RhoA/SRF signaling [16], [22]. In addition to its growth-inhibiting properties, CREG can also act as a negative regulator of VSMC migration by depressing the activation of MMP-9[14]. We also find CREG expression might activate multiple signaling pathways including PI3K/AKT, MAPK and M6P/IGF2R to maintenance of vascular homeostasis[23]. Recently, we observed CREG is a novel of adventitial fibroblasts phenotypic modulator in a p38MAPK-dependent manner[22].

The speed of reendothelialization of an injured artery is an important determinant of neointimal proliferation[24]. Endothelial damage and/or dysfunction can induce local subacute or late thrombosis, leading to blockade of the coronary artery. It can also render VSMCs to be directly exposed to various mitogens in the serum that stimulate VSMC proliferation[25]. Our previous studies showed that CREG can promote wound healing and repair the endothelium of injured arteries. We also found that the level of CREG was reduced in the atherosclerotic artery wall compared with the level in healthy vessels, especially in the endothelium[15], [26]. We have reported that CREG induces endothelial cell migration by activating the integrin-linked kinase/AKT/mTOR/VEGF_165_ signaling pathway and protects endothelial cell apoptosis by activating the VEGF/PI3K/AKT signaling pathway [27], [28]. These data indicate that CREG may play a protective role in re-endothelialization and wound healing after vascular injury. In this study, we showed that the early endothelialization rate was significantly higher in the CREGES group after stent deployment, and similar inflammatory and injury scores observed in SES and CREGES groups vs. BMS group. Regarding DES evaluation, a significant correlation is found between the degree of vascular injury and the inflammatory score [29]. Some preclinical studies have failed to show any significant differences between DES and BMS in the inflammatory score and injury score when 1∶1 to 1∶2 balloon artery ratio is chosen [30], [31]. In current study, the vascular injury and inflammatory caused by stenting, as judged by the vascular injury score and inflammatory score were controlled at the same levels. Under these experimental conditions we observed accelerated endothelialization corresponded to reduced neointimal proliferation, suggesting that the enhancement of reendothelialization may contribute to the suppression of neointima formation by CREGES. Although after 4 weeks, there were no significant differences neointimal formation between SES and CREGES. But, we demonstrated that CREGES significantly enhances re-endothelialization. Since re-endothelialization was negatively correlated with intimal proliferation, CREG administration was a promising new approach to improve long-term results after stenting. This suggested that CREG may prevent restenosis and had a theoretical advantage over other antiproliferative agents, such as sirolimus in that CREG may not delay regrowth of the endothelium.

The current DES systems are composed of three components: a stent, drug, and carrier matrix. Immunosuppressive or antiproliferative drugs are applied to the stent struts to prevent in-stent restenosis. Regarding the carrier matrix, a polymer coating is used in the majority of the current DES because it possesses a desirable drug-releasing property, thereby markedly inhibiting the restenotic process. However, the polymer coating is subject to mechanical damage or deformity at the time of stent implantation [32]. DES polymers have also raised concerns, such as the provoking of allergic reactions, prolonged inflammation and foreign body responses. These biological reactions may cause stent thrombosis and sudden cardiac death [6], [33]. Given these concerns, efforts to develop a new DES and reduce the risks imposed by polymers are underway. Among such efforts is the use of porous stent surfaces as a new drug delivery technology, which may offer both desirable drug elution properties and favorable clinical outcomes. In the current study, a novel polymer-free stent with a nanoporous surface was used as the platform. The stents underwent modification to form in situ micropores on the surface. The selectively nanoporous surface allows the antiproliferative drug to absorb into the stent surface without the use of a polymer. The present study showed that the CREGES offered desirable drug-elution properties without the use of polymers, which may translate into an improved safety profile for the next-generation of DES.

### Limitations

First, because this was an animal study, the results obtained are difficult to directly correlate with clinical trials. In present study experimental stents were deployed in normal porcine coronary arteries, quite different from atherosclerotic human coronary arteries. This experimental model addresses the modulation of neointima formation in a normal vessel, not the process of remodeling of a stenotic coronary vessel. However, the stages of healing are remarkably similar between humans and pigs. The porcine coronary stent model has proven a popular model for the evaluation of drug-eluting stents and is a well-accepted standard. Second, different drugs were present in the polymer-free stents and the polymer-based stents, which may have resulted in the elicitation of different vascular responses. Third, the follow-up time was relatively short; thus, long-term studies need to be carried out.

## Supporting Information

Figure S1 **The effects of nanoporous stents (NS) without CREG on neointimal formation after stent implantation.** 9 NS were implanted into the right and left coronary arteries of 3 domestic pigs (3 stents per animal). The arteries were harvested at 4 weeks and stained with hematoxylin and eosin (A). The areas of neointima in the cross sections of the artery were measured morphmetrically and plotted (B). n = 9 for NS group, n = 12 for BMS, SES and CREGES group. *P<0.05 as compared to BMS, ^#^ P<0.05 as compared to NS.
(TIF)
