# Supplementary figures and images for: Nanoporous CREG-Eluting Stent Attenuates In-Stent Neointimal Formation in Porcine Coronary Arteries
Source: PLoS One. 2013 Apr 3;8(4):e60735. doi: 10.1371/journal.pone.0060735 (PMC3616099; doi:10.1371/journal.pone.0060735)

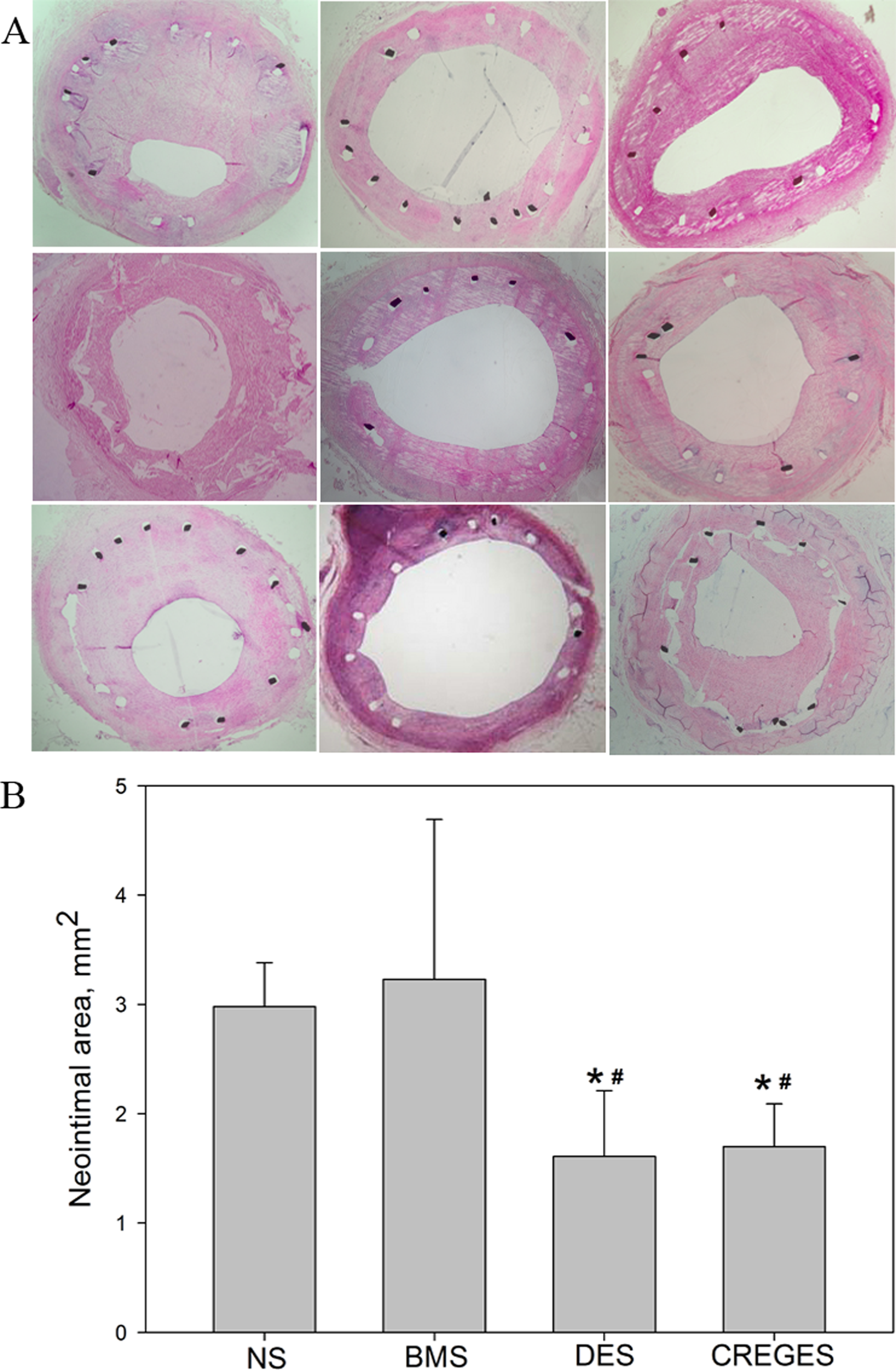

Supplement: Figure S1 — The effects of nanoporous stents (NS) without CREG on neointimal formation after stent implantation. 9 NS were implanted into the right and left coronary arteries of 3 domestic pigs (3 stents per animal). The arteries were harvested at 4 weeks and stained with hematoxylin and eosin (A). The areas of neointima in the cross sections of the artery were measured morphmetrically and plotted (B). n = 9 for NS group, n = 12 for BMS, SES and CREGES group. *P<0.05 as compared to BMS, # P<0.05 as compared to NS. (TIF) [file pone.0060735.s001.tif]
